# Presence of Cleaved Synaptosomal-Associated Protein-25 and Decrease of Purinergic Receptors P2X3 in the Bladder Urothelium Influence Efficacy of Botulinum Toxin Treatment for Overactive Bladder Syndrome

**DOI:** 10.1371/journal.pone.0134803

**Published:** 2015-08-04

**Authors:** Hsin-Tzu Liu, Sung-Ho Chen, Michael B. Chancellor, Hann-Chorng Kuo

**Affiliations:** 1 PhD Program in Pharmacology and Toxicology, Tzu Chi University, Hualien, Taiwan; 2 Voiding Dysfunction Therapeutic Center, Department of Medical Research, Tzu Chi General Hospital, Hualien, Taiwan; 3 Department of Pharmacology, Tzu Chi University, Hualien, Taiwan; 4 Department of Urology, William Beaumont Hospital Research Institute, Oakland University William Beaumont School of Medicine, Royal Oak, Michigan, United States of America; 5 Department of Urology, Buddhist Tzu Chi General Hospital and Tzu Chi University, Hualien, Taiwan; Cedars-Sinai Medical Center, UNITED STATES

## Abstract

**Objectives:**

To evaluate whether botulinum toxin A (BoNT-A) injection and Lipotoxin (liposomes with 200 U of BoNT-A) instillation target different proteins, including P2X3, synaptic vesicle glycoprotein 2A, and SNAP-25, in the bladder mucosa, leading to different treatment outcomes.

**Materials and Methods:**

This was a retrospective study performed in a tertiary teaching hospital. We evaluated the clinical results of 27 OAB patients treated with intravesical BoNT-A injection (*n* = 16) or Lipotoxin instillation (*n* = 11). Seven controls were treated with saline. Patients were injected with 100 U of BoNT-A or Lipotoxinin a single intravesical instillation. The patients enrolled in this study all had bladder biopsies performed at baseline and one month after BoNT-A therapy. Treatment outcome was measured by the decreases in urgency and frequency episodes at 1 month. The functional protein expressions in the urothelium were measured at baseline and after 1 month. The Wilcoxon signed-rank test and ordinal logistic regression were used to compare the treatment outcomes.

**Results:**

Both BoNT-A injection and Lipotoxin instillation treatments effectively decreased the frequency of urgency episodes in OAB patients. Lipotoxin instillation did not increase post-void residual volume. BoNT-A injection effectively cleaved SNAP-25 (*p* < 0.01). Liposome encapsulated BoNT-A decreased urothelial P2X3 expression in the five responders (*p* = 0.04), while SNAP-25 was not significantly cleaved.

**Conclusions:**

The results of this study provide a possible mechanism for the therapeutic effects of BoNT-A for the treatment of OAB via different treatment forms. BoNT-A and Lipotoxin treatments effectively decreased the frequency of urgency episodes in patients with OAB.

## Introduction

Intravesical onabotulinumtoxin A (BoNT-A) injection decreases urgency and urgency urinary incontinence (UUI) in patients with overactive bladder syndrome (OAB) [1–3]. This treatment is widely used among OAB patients refractory to antimuscarinic therapy and has gained standing in countries in North America and Europe. Recent investigations focused on intravesical instillation of liposome-encapsulated BoNT-A (Lipotoxin) revealed the therapeutic effect of decreased frequency and urgency episodes in OAB patients, but UUI episodes were not decreased [4,5].

BoNT-A injections in bladders of patients with OAB result in increased bladder capacity, decreased detrusor contractility, and reductions in frequency and urgency episodes [6,7]. However, the post-void residual (PVR) volume increased, which could place frail elderly OAB patients at risk of a large PVR and urinary retention [8]. On the other hand, Lipotoxin instillation results in a reduction of frequency and urgency episodes but not UUI, and no alteration in detrusor contractility or increase in PVR volume were noted [4,5]. It is possible that BoNT-A can only be delivered by liposomes to the superficial urothelial layer and not deep into the suburothelial tissue or detrusor layer.

BoNT-A binds to the high-affinity receptor synaptic vesicle protein 2 (SV2). After internalization into the cytoplasm, BoNT-A cleaves specific sites of synaptosomal-associated protein-25 (SNAP-25) to inhibit the exocytosis of neurotransmitters from the nerve terminals [9]. The therapeutic effect of BoNT-A is demonstrated by immunohistochemical staining of the cleavage product, cleaved SNAP-25 (cSNAP-25) [10]. In addition, up-regulation of the purinergic receptor P2X3 occurs in OAB bladders along with neurogenic detrusor overactivity [11,12]. After injection of BoNT-A into the detrusor, the expression of P2X3 receptors decreases significantly, as does adenosine triphosphate (ATP) release [13,14]. The biological effects of BoNT-A seem to be both efferent and afferent via modulation of ATP release and inhibition of acetylcholine release [15].

In this study, we evaluated the expressions of P2X3 receptors as well as SNAP-25 and cSNAP-25 in the urothelium and explored the different mechanisms of action of BoNT-A injection and Lipotoxin instillation. We also analyzed the effectiveness of OAB treatment. The results provide evidence of the therapeutic effects of BoNT-A in OAB through different modes of treatment.

## Materials and Methods

This was a retrospective study performed in a tertiary teaching hospital. Patients with OAB who had been enrolled in clinical trials of intravesical BoNT-A injection (clinicaltrial.gov identifier: NCT01657409) were retrospectively included in this study. The OAB patients who had received Lipotoxin intravescal instillation (clinicaltrial.gov identifier: NCT01167257) were the same subjects as included in our previous study [4]. However, patients who refused to undergo bladder biopsy examination were excluded in this study. OAB patients refractory to antimuscarinic treatment for more than three months were included in order to evaluate the therapeutic safety and efficacy of intravesical BoNT-A injection or Lipotoxin instillation. The patients included had completed antimuscarinic treatments, voiding diaries and urodynamic studies. Patients who had received placebo treatment in the Lipotoxin clinical trial served as the controls. Furthermore, the patients enrolled in this study all had bladder biopsies performed at baseline and one month after BoNT-A treatment. Bladder biopsies were performed by cystoscopy, and only bladder mucosa tissues (including the urothelium, lamina propria and a few discontinuous muscularis mucosa) were obtained.

This study was approved by the Institutional Review Board and Ethics Committee of Buddhist Tzu Chi General Hospital (IRB 098–86). Written informed consent was obtained from every patient after informing them of the potential adverse events related to BoNT-A injection or intravesical Lipotoxin instillation.

### BoNT-A injection and Lipotoxin instillation techniques

The patients selected for this study were treated with detrusor intravesical injection of BoNT-A at 20 sites using a 23-gauge needle in a rigid cystoscopic injection instrument (22 Fr, Richard-Wolf, Knittlingen, Germany). One-hundred units of BoNT-A were reconstituted to 10 ml with normal saline. All procedures and after-injection care were in accordance with previous reports [1,6–8]. Lipotoxin was made by mixing liposomes (80 mg/40 ml) with 200 U of BoNT-A (BOTOX, Allergan, Irvine, CA, USA) in 10 ml of distilled water for use as a single intravesical instillation. The control patients were instilled with 50 ml of normal saline solution [4,5].

The OAB patients were evaluated for changes in frequency, urgency, and UUI episodes and kept a voiding diary for this purpose; the overactive bladder symptom score and urgency severity scale were also assessed. Functional bladder capacity, maximum flow rate, and voided and PVR volumes were also recorded. Patients who had decreases in urgency and UUI episodes for three consecutive days were considered responders. A research assistant who was unaware of the treatment group assignments measured the outcome variables.

### Analysis of SNAP-25 and cSNAP-25 proteins in the bladder mucosa

**Fig 1:**
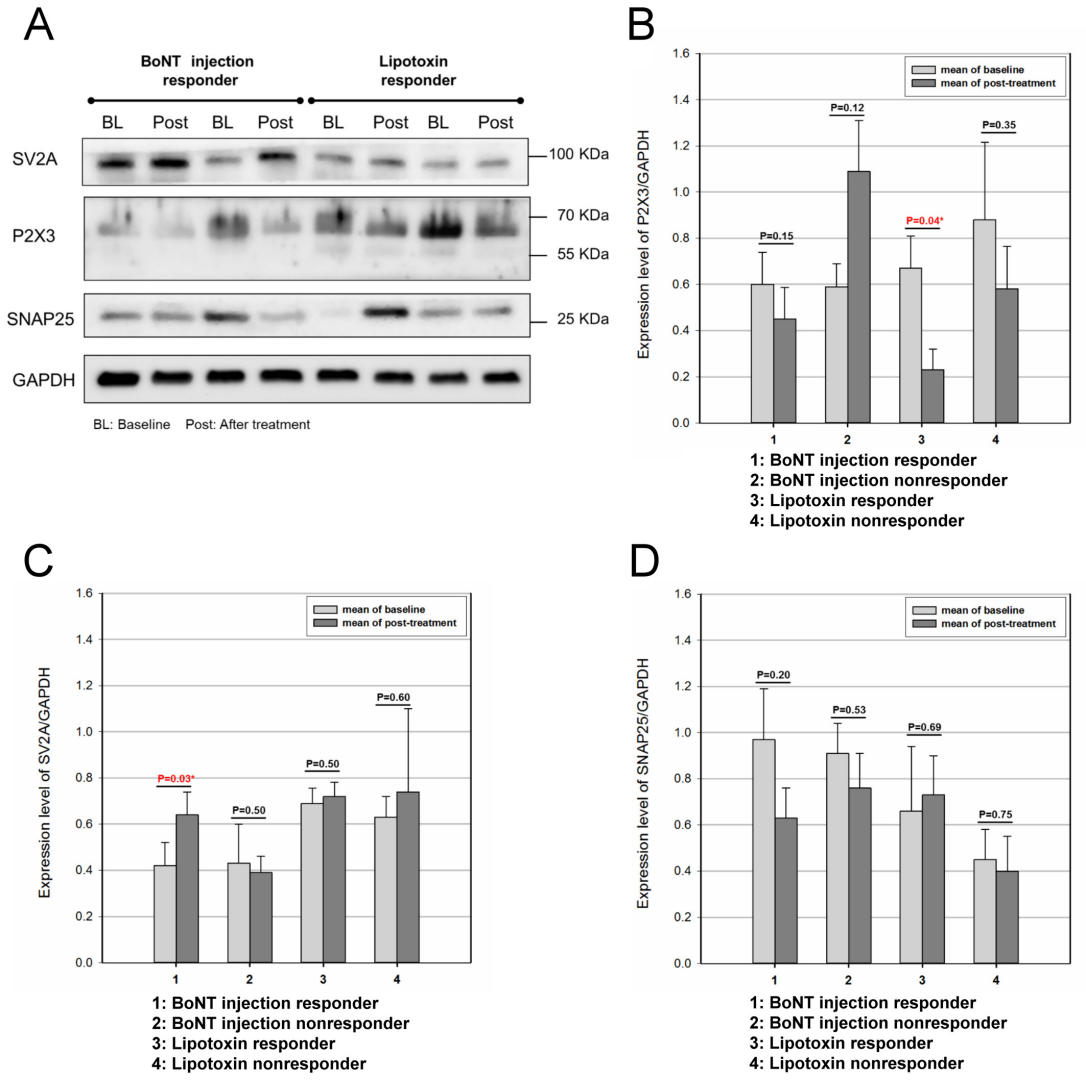
Expression of SV2A, P2X3 receptors, and SNAP-25 in the bladder mucosa of OAB patients at baseline and one month after BoNT-A injection or Lipotoxin treatment. (A) Immunoblot analysis of SV2A (95 KDa), P2X3 (66 KDa), and SNAP-25 (26 KDa) expression in OAB patients in response to BoNT-A injection or Lipotoxin treatment. (B, C, and D) Relative expression changes of P2X3, SV2A receptors, and SNAP-25, respectively, in OAB patients after BoNT-A injection or Lipotoxin treatment. There were no significant differences in any of the proteins between the BoNT-A responders and non-responders, or between the Lipotoxin responders and non-responders. GAPDH = glyceraldehyde phosphate dehydrogenase.

Functional protein analysis of SNAP-25 and cSNAP-25 was performed on the bladder tissues biopsied at baseline and at 1month post-treatment. Urinary bladder specimens were fixed and processed for immunohistochemical analyses as previously described [4]. Four sections per specimen were cut using a cryostat to a thickness of 10 μm. Sections were incubated overnight at 4°C with an antibody against human SNAP-25 (Abcam, Cambridge, UK) or human cleaved SNAP-25 (GeneTex, Irvine, CA, USA). Streptavidin-biotin peroxidase complex (ABC) technology (super sensitive IHC detection systems; BioGenex Laboratories, Fremont, CA, USA) was used. The negative controls were processed at the same time using the same procedures but omitting the primary antibody. The images were obtained using a digital imaging system (Carl Zeiss, Oberkochen, Germany). Analysis of the expression of cSNAP-25 was based on the density of the fibers with immunoreactivity to the cSNAP-25-specific antibody. The fibers were measured in five blocks (1500 μm^2^ in total).The total length of the immunoreactive fibers in these five blocks was normalized to a constant of 100 μm^2^ (Fig 1).

### Analyses of urothelial SV2A, SNAP-25, and P2X3 receptor expressions

Bladder specimens were homogenized in protein extraction solution (T-PER; Thermo Scientific, USA) with proteinase and phosphatase inhibitor cocktail (Roche Applied Science, Penzberg, Germany). The standard techniques for immunoblotting were followed. Briefly, 10 μg of bladder protein extract were electrophoresed at a constant voltage of 150 V for 2 h and were then transferred to Immune-Blot PVDF Membranes (Bio-Rad, Hercules, CA, USA). The membranes were immunoblotted overnight at 4°C with the primary antibody. The antibodies used were as follows: rabbit anti-human SV2A antibody (Abcam, Cambridge, UK), goat anti-human SNAP-25 antibody (Abcam, Cambridge, UK), and rabbit anti-human P2X3 antibody (Santa Cruz Biotechnology, USA). After washing the membranes, they were incubated with a secondary antibody for 2 h at room temperature. The amount of glyceraldehyde phosphate dehydrogenase was calculated as the internal control. Quantitative analysis was performed using TotalLab Quant software V13.2 (TotalLab Ltd. UK).

### Statistical analysis

All data were expressed as the mean ± standard error of the mean. The Wilcoxon signed-rank test was used to evaluate the differences in variables between baseline values and post-treatment values. The ordinal logistic regression method was used to analyse the changes in USS and GRA after treatment. A *p* value of less than 0.05 was considered to indicate statistical significance. Statistical analyses were performed using SPSS statistical software (version 12.0, SPSS Inc., Chicago, IL, USA).

## Results

This study included 16 OAB patients (nine men and seven women; mean age, 67.3 ± 10.6 years) who received intravesical BoNT-A 100 U injection and 11 OAB patients (five men and six women; mean age, 67.3 ± 15.2 years) who were treated with intravesical Lipotoxin instillation. The placebo control group included 7 patients (five men and two women; mean age, 72.7 ± 6.9 years) who received normal saline treatment in the Lipotoxin trial. All patients had wet OAB, urodynamically proven detrusor overactivity and no neurogenic detrusor overactivity or urinary tract infection at enrollment.

### Clinical treatment outcomes

Table 1 shows the clinical results of BoNT-A injection, Lipotoxin instillation, and normal saline instillation. The frequency episodes, urgency episodes averaged over three days and overactive bladder symptom score improved significantly in the BoNT-A injection and Lipotoxin instillation groups, but not in the control group. Changes in global response assessment were noted in all three groups. Nonetheless, functional bladder capacity and PVR increased only in the BoNT-A injection group. Overall, ten (62.5%) patients in the BoNT-A injection group, five (45.5%) patients in the Lipotoxin instillation group, and none in the control group responded to the treatments.

**Table 1:** Clinical and urine flow variables at baseline and one month after BoNT-A injection or Lipotoxin instillation.

		BoNT-A injection (*n* = 16)	Lipotoxin instillation (*n* = 11)	Normal saline instillation (*n* = 7)
**Frequency/3 days**	Baseline	36.3 ± 7.4	37.4 ± 11.9	28.0 ± 7.8
	1 month	29.6 ± 6.7*	28.7 ± 7.7*	25.7 ± 6.2
**Urgency/3 days**	Baseline	33.1 ± 8.6	30.8 ± 15.5	23.3 ± 12.5
	1 month	20.3 ± 12.6*	19.7 ± 12.0*	23.7 ± 9.4
**UUI/3 days**	Baseline	9.4 ± 12.9	4.5± 7.2*	7.1 ± 7.3
	1 month	4.5± 7.2*	12.5 ± 4.50	7.2 ± 10.5
**OABSS**	Baseline	11.7 ± 2.1	11.1 ± 2.1	10.7 ± 1.7
	1 month	8.7 ± 3.3*	9.2 ± 3.2*	9.7 ± 3.1
**FBC (ml)**	Baseline	293.3 ± 130.7	384.6 ± 119.5	311.4 ± 96.7
	1 month	373.3 ± 214.7*	342.7 ± 183.8	327.1 ± 152.2
**Qmax (ml/s)**	Baseline	15.8 ± 8.0	17.0 ± 9.5	13.7 ± 3.6
	1 month	14.2 ± 6.7	18.0 ± 9.9	12.6 ± 7.0
**Voided volume (ml)**	Baseline	216.8 ±102.5	269.8 ± 122.7	201.0 ± 50.5
	1 month	230.1± 160.8	242.5 ± 132.9	206.6 ± 79.1
**PVR (ml)**	Baseline	11.2 ± 15.6	55.5 ± 39.3	9.6 ± 15
	1 month	142.6 ± 56.1*	51.6 ± 40.0	25.0 ± 41.1
**GRA**	1 month	1.5 ± 1.5*	1.5 ± 1.0*	1.3 ± 1.1*
**Responder (%)**	1 month	10 (62.5%)	5 (45.5%)	0

*: Significant difference between baseline and one month after treatment.

Data are expressed as means ± standard deviations.

UUI = urgency urinary incontinence, OABSS = overactive bladder symptom score, FBC = functional bladder capacity, Qmax = maximum flow rate, PVR = post-void residual, GRA = global response assessment.

### SNAP-25 and cSNAP-25 after BoNT-A injection

**Fig 2:**
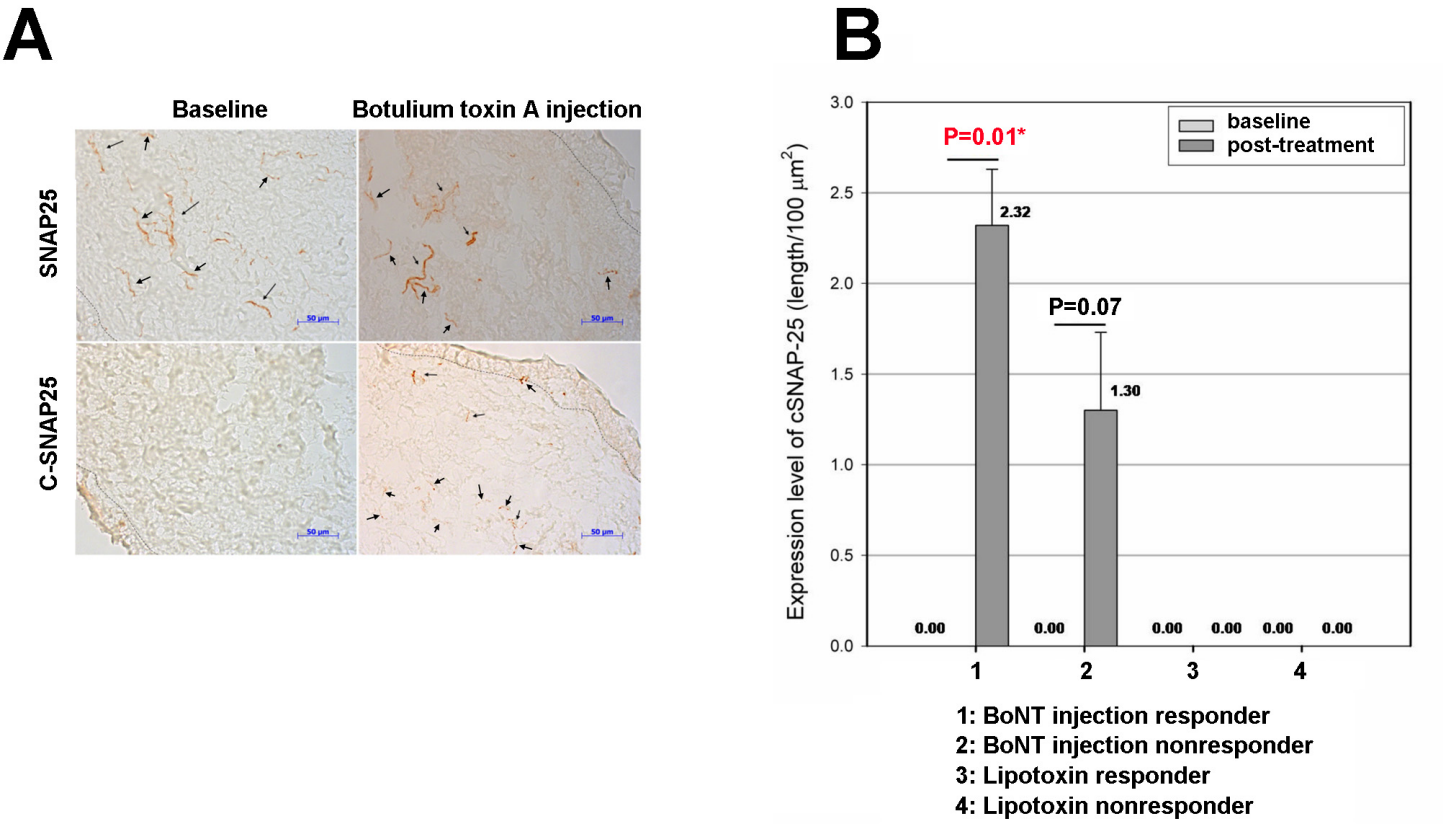
Immunohistochemistry staining of SNAP-25 and cleaved SNAP-25 (cSNAP-25) in bladder mucosa samples of OAB patients following BoNT-A injection. (A) SNAP-25 expression (arrows) was detected in patients at baseline and after BoNT-A treatment. cSNAP-25 was not detected at baseline but was present in the suburothelium of OAB bladders after BoNT-A injection. (B) Expression of cleaved SNAP-25 in patients receiving BoNT-A injections or lipotoxin treatment. There was no significant difference in cSNAP-25 expression between the BoNT-A responders and nonresponders. cSNAP-25 was not detected in the OAB patients who underwent Lipotoxin treatment, whether at baseline or after treatment.

SNAP-25 expression occurs in the suburothelium of the bladder [4]. In the immunohistochemistry analysis, SNAP-25 was expressed at baseline and one month after BoNT-A injection or Lipotoxin treatment. cSNAP-25 was not detected at baseline in the bladder of any OAB patient but was present in the suburothelium of bladders of patients with OAB after BoNT-A injection (Fig 2). However, cSNAP-25 was not detected in any OAB bladder after Lipotoxin instillation (Table 2).

**Table 2:** Changes inP2X3, SV2A, SNAP-25, and cSNAP-25 in controls and OAB patients receiving BoNT-A injection or Lipotoxin or normal saline instillation at baseline and one month after treatment.

		BoNT-A injection (*n* = 16)	Lipotoxin instillation (*n* = 11)	Lipotoxin instillation (*n* = 11)
P2X3*	Baseline	0.54 ± 0.10	0.62 ± 0.13	0.86 ± 0.31
	1 month	0.60 ± 0.13	0.44 ± 0.11	0.78 ± 0.28
		*p* = 0.95	*p* = 0.14	*p* = 0.50
SV2A*	Baseline	0.43 ± 0.09	0.63 ± 0.15	0.52 ± 0.11
	1 month	0.55 ± 0.07	0.56 ± 0.07	0.82 ± 0.24
		*p* = 0.04^**‡**^	*p* = 1.00	*p* = 0.08
SNAP-25*	Baseline	0.95 ± 0.14	0.59 ± 0.15	0.32 ± 0.07
	1 month	0.68 ± 0.10	0.52 ± 0.10	0.54 ± 0.13
		*p* = 0.33	*p* = 0.82	*p* = 0.06
cSNAP-25^†^	Baseline	0	0	0
	1 month	1.94 ± 0.27	0	0
		*p* < 0.01^**‡**^		

Data are expressed as means ± standard error of the mean.

*: Detected by protein immunoblotting.

^†^: Detected by immunohistochemistry.

^**‡**^: Significant difference between baseline and one month after treatment.

BoNT-A = onabotulinumtoxinA, SV2A = synaptic vesicle protein 2A, SNAP-25 = synaptosomal-associated protein-25, cSNAP-25 = cleaved synaptosomal-associated protein-25.

Table 3 lists the changes in SNAP-25 and cSNAP-25 in the BoNT-A injection and Lipotoxin instillation responders and non-responders. The change in SNAP-25 between responders and non-responders was not statistically significant (*p* = 0.94). cSNAP-25 expression was present only in the patients who received BoNT-A injections (*p* < 0.01), and not in patients who received Lipotoxin instillation. There was no significant difference in the change of cSNAP-25 between responders and non-responders to BoNT-A injection (*p* = 0.82).

**Table 3:** Changes inP2X3, SV2A, SNAP-25, and cSNAP-25 expressions between responders and non-responders to BoNT-A injection or Lipotoxin instillation at baseline and one month after treatment.

		BoNT-A injection		Lipotoxin instillation	
		Responder (*n* = 10)	Non-responder (*n* = 6)	Responder (*n* = 5)	Non-responder (*n* = 6)
**P2X3** *	Baseline	0.61 ± 0.14	0.42 ± 0.11	0.61 ± 0.21	0.63 ± 0.18
	1 month	0.47 ± 0.13	0.82 ± 0.26	0.39 ± 0.13	0.50 ± 0.17
		*p* = 0.17	*p* = 0.12	*p* = 0.04^**‡**^	*p* = 0.53
**SV2A** *	Baseline	0.42 ± 0.10	0.43 ± 0.17	0.49 ± 0.13	0.81 ± 0.30
	1 month	0.64 ± 0.10	0.39 ± 0.07	0.49 ± 0.10	0.65 ± 0.10
		*p* = 0.03^**‡**^	*p* = 0.50	*p* = 1.00	*p* = 1.00
**SNAP-25** *	Baseline	0.97 ± 0.22	0.91 ± 0.13	0.76 ± 0.27	0.45 ± 0.13
	1 month	0.63 ± 0.13	0.76 ± 0.15	0.68 ± 0.08	0.40 ± 0.16
		*p* = 0.20	*p* = 0.53	*p* = 1.00	*p* = 0.75
**cSNAP-25** ^**†**^	Baseline	0	0	0	0
	1 month	2.32 ± 0.31	1.30 ± 0.43	0	0
		*p* < 0.01^**‡**^	*p* = 0.07		

Data are expressed as means ± standard error of the mean.

*: Detected by protein immunoblotting.

^†^: Detected by immunohistochemistry.

^**‡**^: Significant difference between baseline and one month after treatment.

BoNT-A = onabotulinumtoxinA, SNAP-25 = synaptosomal-associated protein-25, cSNAP-25 = cleaved synaptosomal-associated protein-25.

### SV2A and P2X3 receptor expressions after BoNT-A treatment

After 1month of BoNT-A treatment, the SV2A receptor level increased significantly in the BoNT-A injection group (*p* = 0.04) but not in the Lipotoxin instillation group (*p* = 1.0) (Table 2). According to the definition of responders to BoNT-A injection, ten patients responded with SV2A receptor levels higher than at baseline (*p* = 0.03), while the SV2A level did not change in the six non-responders (*p* = 0.50) (Table 3).

Among the 16 patients who received BoNT-A injections, 11 who received Lipotoxin instillation, and seven control patients, the P2X3 receptor expression did not differ between baseline and 1 month after treatment (*p* = 0.95, 0.14, and 0.50, respectively) (Table 2). According to the improvement in frequency episodes averaged over 3 days and urgency episodes averaged over 3 days among the 11 Lipotoxin instillation group patients, five patients experienced frequency and urgency reductions and were considered responders. After Lipotoxin therapy, the P2X3 expression decreased significantly in the five responders (*p* = 0.04), but no significant change was noted in the six non-responders (*p* = 0.53) (Table 3).

## Discussion

This study demonstrated that BoNT-A injection can decrease urgency and UUI episodes, and a trend of a decrease in P2X3 expression in responders was observed. This treatment can effectively cleave SNAP-25 to cSNAP-25; thus, the therapeutic effects could involve both sensory and motor mechanisms. On the other hand, Lipotoxin instillation only delivered BoNT-A to the superficial urothelium; therefore, urothelial P2X3 expression was decreased but cSNAP-25 could not be detected in the suburothelium by immunohistochemical staining. This evidence supports the clinical therapeutic results that BoNT-A injection increases the bladder capacity and decreases detrusor contractility, while Lipotoxin instillation results in decreased frequency and urgency episodes but not UUI episodes [4]. Nevertheless, Lipotoxin instillation can avoid the adverse events that occur after BoNT-A injection, such as acute urinary retention, large PVR, and urinary tract infection [1–3].

The mechanism of effective BoNT-A treatment in OAB is successful cleavage of SNAP-25 at the nerve terminals and prevention of neurotransmitter exocytosis in the vesicles [9]. Thus, the post-synaptic receptors cannot be fully activated due to a lack of adequate transmitters, and the desired functional impairment is achieved [16]. The immunoreactivity of SNAP-25 is observed in the parasympathetic nerves of the detrusor in human bladders [17,18]. cSNAP-25 is abundant in the cholinergic nerves after BoNT-A detrusor injection in patients with OAB [18,19]. The expression of SNAP-25 in the bladder wall decreases after intravesical BoNT-A treatment in rats [20]. However, recent investigations revealed that the total SNAP-25 might not change, but the presence of cSNAP-25 indicates the mechanistic effects of BoNT-A on the nerve fibers in the bladder wall and can be used as a marker of BoNT-A action [9,21].

SV2A is required for normal calcium-evoked neurosecretion [22]. In the human urinary bladder, SV2A receptors are found in the urothelium, suburothelium, and detrusor muscle [4,23]. A recent investigation demonstrated that patients with idiopathic detrusor overactivity and those with painful bladder syndrome both have more SV2A nerve fibers than the controls [23]. The results of this study showed that SV2A receptor expression increased significantly in OAB patients after BoNT-A injection, but no change was seen in patients who received Lipotoxin instillation. There is emerging evidence that BoNT-A blocks neuropeptide release from the afferent nerves and exocytosis of acetylcholine from efferent nerves [24]. SV2A is essential for neurosecretion. Based on this evidence, we hypothesized that the increase in SV2A expression after BoNT-A injection could be a biofeedback effect in order to compensate for decreases in neuropeptide secretion.

BoNT-A is a neurotoxin with a high molecular weight of 150 kilo Daltons, It is difficult to access the submucosal nerve plexus with the formal use of saline as a vehicle without direct injection to pass the urothelial barrier. Since 2002, Chancellor et al. have been successful in developing intravesical liposomes for interstitial cystitis/painful bladder syndrome (IC/PBS). They discovered and translated an intravesical instillation of liposome formulation that can coat and protect the bladder [25].The therapeutic effects of liposomes on IC/BPS were evident [26]. Fraser et al. reported a physiological effect of intravesical liposome alone in a hyperactive bladder model that involved the use of protamine and KCl [27]. A recent study demonstrated that liposome encapsulation of BoNT-A is able to improve the rate of bladder uptake of BoNT-A without breaking down the bladder permeability barrier [20].The changes in SNAP25 and CGRP immunoreactivity were significant in Lipotoxin-treated rat bladders but not in those treated with liposomes or BoNT-A instillation alone. This method overcomes the need for physical or chemical trauma to break the urothelial barrier before delivering BoNT-A into the bladder.

Injection of BoNT-A directly into the suburothelial space reduces detrusor contractility, urgency, and UUI episodes [1,6,7]. Both afferent and efferent mechanisms appear to play roles in the physiological effects of BoNT-A treatment for OAB [28]. On the other hand, Lipotoxin instillation might only deliver BoNT-A to the more superficial urothelial layer. Although urgency and frequency episodes are reduced after Lipotoxin instillation, detrusor contractility is barely affected [4,5]. It is possible that the physiologies of the therapeutic effects are different between the two treatment modalities. BoNT-A injection decreases the expressions of functional proteins in the suburothelium and detrusor, while Lipotoxin affects only the most superficial urothelial cells.

In this study, we also found that urothelial P2X3 receptors decreased significantly in responders after Lipotoxin instillation. Recently, the ATP-gated ion channel P2X3 has gained special attention. P2X3 receptors are critical for afferent pathways mediating sensory afferent excitation and controlling bladder volume [29]. P2X3 immunoreactive nerves are found abundantly throughout the urothelium and muscle layer in human bladders [28, 30]. Up-regulation of P2X3 occurs in neurogenic detrusor overactivity and OAB [10,11]. BoNT-A significantly attenuates bladder afferent nerve firing, inhibits ATP release from the urothelium, and has an efferent function in overactive bladders [12,31]. BoNT-A treatment of OAB patients results in blockade of ATP co-released with acetylcholine from muscle efferent fibers, causing a reduction in the urgency sensation [32].

P2X3 immunoreactive fibers in the suburothelium and detrusor were found to decrease significantly after BoNT-A injection in patients with OAB as well as NDO [13, 14, 28]. However, the urothelial expression of P2X3 is not significantly decreased in these patients after BoNT-A injection, suggesting that the effect on the sensory receptors was lower in the urothelium than in the suburothelium and detrusor using the injection technique to deliver BoNT-A into the bladder wall [28]. In contrast, using liposomes to carry BoNT-A across the urothelial barrier enables BoNT-A to directly act on the synaptic vesicles in the urothelial cells, and hence the P2X3 expression in the urothelium was significantly decreased in the responders. The exact mechanism of the decrease in P2X3 receptors after BoNT-A treatment has not been clarified yet. Previous study has shown that BoNT-A treatment does not decrease the total neuronal number, implying more complex and multiple mechanisms of action of BoNT-A on sensory fibers [28].

In a rat model, SNAP-25 decreases after BoNT-A injection [20], while the density of SNAP-25 protein appears not to change in humans after BoNT-A injection. Nonetheless, investigators recently reported that a limited amount of SNAP-25 was cleaved by BoNT-A injection in patients with myelomeningocele [19]. It is likely that detrusor injection of 100 U of BoNT-A might not extensively cleave all SNAP-25 in the bladder walls of OAB patients. Nevertheless, the presence of cSNAP-25 after BoNT-A injection proves that BoNT-A injection effectively cleaves SNAP-25 and has a therapeutic effect in human patients with OAB. The lack of cSNAP-25 in the Lipotoxin instillation group further demonstrated that BoNT-A cannot be delivered as deeply by liposomes as by injection. It is possible that the BoNT-A protein being carried across the urothelium and down to the suburothelial nerve fibers is limited, so that SNAP-25 protein in the suburothelium was not found to have decreased 1 month after treatment.

This study was limited by the fact that the case number was small and patients were not enrolled in the same clinical trial. However, the laboratory work was performed at the same time and by the same investigators, who were not aware of the patients’ allocations to treatment groups. In addition, the dose of BoNT-A was 100 U onabotulinumtoxin A for injection and 200 U for Lipotoxin instillation. This discrepancy in dose might result in different therapeutic outcomes and bias. However, in this study, we for the first time investigated whether liposomes can act as a vehicle to carry BoNT-A protein across the urothelial barrier. The purpose of the study was to prove this concept and technical availability. Whether 200 U onabotulinumtoxin A in Lipotoxin instillation could have a therapeutic efficacy comparable to that of 100 U onabotulinumtoxin A injection needs further clinical investigation.

We demonstrated that BoNT-A injection effectively cleaves SNAP-25, whereas Lipotoxin decreases P2X3 expression in the urothelium of responders but does not significantly cleave SNAP-25 in the suburothelium. Both treatments effectively decreased the frequency and urgency episodes in patients with OAB, and Lipotoxin instillation did not increase PVR after treatment.
